# Acceptance, experience, and feedback for supplemental screening in dense breasts among women participating in the BRAID trial

**DOI:** 10.1186/s13244-025-02170-8

**Published:** 2026-01-16

**Authors:** Iris Allajbeu, Kate R. Charnley, Yuyin Yang, Johanna Field-Rayner, Kirsten Morris, Nicholas R. Payne, Fleur Kilburn-Toppin, Roido Manavaki, Fiona J. Gilbert

**Affiliations:** 1 Department of Radiology, University of Cambridge School of Clinical Medicine, Cambridge Biomedical Campus, Cambridge, UK; 2 Department of Radiology, Addenbrookes Hospital, Cambridge University Hospitals NHS Foundation Trust, Cambridge, UK; 3 Western Balkans University, School of Clinical Medicine, Tirana, Albania

**Keywords:** Breast, Mass screening, Patient acceptance of healthcare, Patient satisfaction

## Abstract

**Objectives:**

To evaluate patient acceptance and feedback regarding supplemental imaging modalities: automated whole-breast ultrasound (ABUS), contrast-enhanced mammography (CEM), and abbreviated breast MRI (AB-MRI) within the BRAID (Breast Screening: Risk Adaptive Imaging for Density) trial.

**Materials and methods:**

An adapted Testing Morbidities Index questionnaire was utilised to capture participant experiences and perceptions (January-April 2024) related to AB-MRI, ABUS and CEM. Likert-scale questions assessed discomfort, anxiety, and overall satisfaction for each imaging modality, while thematic analysis was applied to free-text patient feedback. Additionally, reasons for withdrawal were recorded for each modality.

**Results:**

Among 159 women providing feedback, 57/159 (35.8%) underwent ABUS, 52/159 (32.7%) CEM, and 50/159 (31.5%) AB-MRI. Acceptability of ABUS, CEM and AB-MRI was rated similarly to mammography by 71/159 (64.8%) of these respondents, with 72/159 (45.3%) considering them superior. Mild-to-moderate discomfort due to breast compression was reported for ABUS and CEM, whereas AB-MRI resulted in the least discomfort. Pre-procedural anxiety was observed across all imaging modalities, particularly with contrast-enhanced techniques; however, experiences were generally well-tolerated. Effective communication and pre-test information reduced anxiety levels, with most participants willing to repeat the procedures. 151/984 (15.3%) withdrawals in BRAID were due to adverse patient experiences, with contrast-enhanced techniques accounting for most of these withdrawals (CEM: 69/151, 45.7%; AB-MRI: 66/151, 43.7%; ABUS: 12/151, 7.9%). The main reasons for withdrawal were unhappiness with the allocated imaging arm and discomfort or anxiety during the procedure.

**Conclusion:**

Supplemental imaging modalities are generally well-accepted by patients with benefit throughout gained by clear communication and preparedness.

**Critical relevance statement:**

Feedback from a subgroup of women participating in the BRAID trial shows that supplemental imaging alongside routine screening is well-accepted. Clear communication and empathetic care further improve acceptance, supporting a shift toward personalised breast cancer screening for women with dense breasts.

**Key Points:**

- Understanding women’s imaging experiences is essential for optimising breast screening practices.
- Acceptability of supplemental imaging was rated similar to or better than mammography by most participants.
- Clear, empathetic communication reduced anxiety and improved experience with contrast-enhanced imaging.

**Graphical Abstract:**

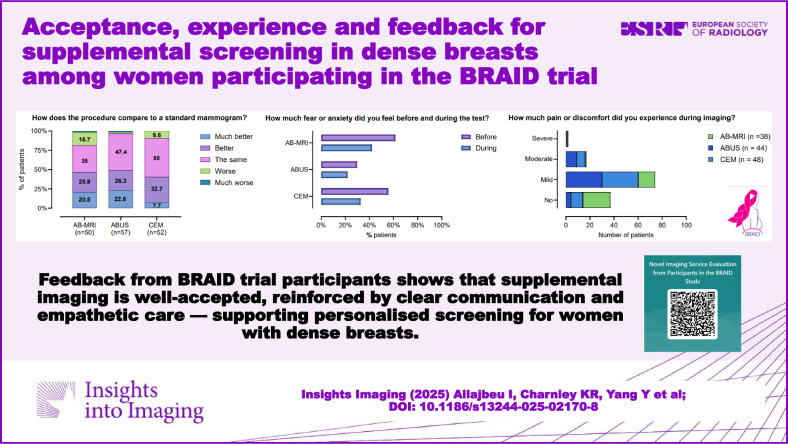

## Introduction

Breast screening is a vital public health initiative for the early detection of breast cancer, the most frequently diagnosed cancer among women. In the United Kingdom (UK), the National Health Service Breast Screening Programme (NHSBSP) invites women aged 50–70 years to undergo standard full-field digital mammography (FFDM) every 3 years [1]. This ‘one-size-fits-all’ approach has significantly contributed to improved breast cancer survival rates, reducing mortality by 20% [2]. However, the sensitivity of mammography is limited in women with dense breasts, where tumours may be masked, leading to cancer underdiagnosis. Women with extremely dense breasts (BI-RADS d) have up to four times the risk of developing breast cancer compared to those with the lowest breast density (BI-RADS a—almost entirely fatty) due to masking and an inherent increased risk [3]. In a large, sequential screening cohort from Cambridge, UK, sensitivity was 59.8% and 51.3% for women with BI-RADS c and d, respectively [4].

In the UK, risk adaptation within NHSBSP is limited to annual magnetic resonance imaging (MRI) alongside mammography for women with a lifetime breast cancer risk of > 30%, and annual mammography for some moderate-risk women [5]. There is growing recognition of the need for risk-stratified screening and supplemental imaging to enhance early breast cancer detection in women with dense breasts, improve the benefit-to-harm ratio for patients, and increase the cost-effectiveness of the current screening programme.

Supplemental imaging techniques, such as automated breast ultrasound (ABUS), contrast-enhanced mammography (CEM) and abbreviated breast (AB)-MRI, can improve breast cancer detection rates (CDR) when used alongside mammography, especially in women with dense breasts, while reducing advanced-stage interval and next-round cancers [5]. These findings have prompted efforts to integrate supplemental imaging into existing screening protocols. To this end, the Breast Screening: Risk Adapted Imaging for Density (BRAID) trial was initiated to evaluate these three supplemental imaging modalities—AB-MRI, ABUS, and CEM—in comparison to standard-of-care mammography. This multicentre randomised controlled trial enrolled 9452 women aged 50–70 years with dense breasts (BI-RADS c or d), offering these supplemental imaging tests twice at 18-month intervals over 3 years. The primary outcome was to determine CDR for each supplemental imaging technique [6].

As the field of breast imaging evolves, understanding patients’ experiences and acceptance of imaging methods is essential for optimising screening practices and improving health outcomes. Patient experience and perceptions play a critical role in determining participation and overall satisfaction with breast screening programmes, with fear of pain, discomfort, and anxiety frequently cited as significant barriers [7, 8]. Cultural beliefs, social norms, logistical challenges, including transportation difficulties and financial constraints, may further hinder participation in routine screening [9–11]. Addressing these barriers through effective communication and tailored education is essential to bridging these gaps [9, 12].

A successful breast cancer screening programme incorporating supplemental imaging should not only be highly accurate but also affordable, widely accessible, and patient-friendly. The BRAID trial provided a unique opportunity to evaluate participant satisfaction and acceptance of supplemental imaging, offering insights into factors influencing patient comfort or willingness to return for future screenings. This service evaluation aimed to assess patient acceptability and satisfaction with the supplemental imaging modalities offered in BRAID versus the standard-of-care, focusing on patient-reported outcomes to gauge the acceptability of these modalities in clinical practice.

## Materials and methods

Between January and April 2024, women aged 50–70 years with dense breasts (BI-RADS c and d) were recruited as part of BRAID (NCT04097366) [6] and invited to participate in this service evaluation. The BRAID trial received ethical approval from a National Research Ethics Committee (NRES Committee London – Surrey; REC no: 19/LO/0350). Additional approval for this service evaluation was provided by Cambridge University Hospitals NHS Foundation Trust (2024/BR-117). All participants provided written informed consent before participation. To assess participant experience and acceptance regarding the supplemental imaging modalities offered within BRAID, we analysed responses from 159 women who underwent ABUS, CEM, or AB-MRI scans twice at 18-month intervals over 3 years as per the BRAID protocol (Supplementary Fig. S1).

A mixed-methods approach, using a tailored questionnaire adapted from the Testing Morbidities Index [13], was employed to assess patient experience. Participants completed the questionnaire immediately following their imaging procedure to facilitate accurate recall of their experience. The questionnaire utilised Likert-scale questions to evaluate key aspects of patient experience, including procedural comfort, emotional response, and overall acceptability, with free-text responses to capture a more detailed understanding of patient perspectives. Withdrawal reasons among the entire BRAID cohort were also analysed by imaging modality, focusing on reasons related to patient experience.

### Questionnaire content

The questionnaire was structured into several domains to assess patient acceptance, satisfaction, and experience (Supplementary file). Each domain included the following components:

- Comparison to standard mammogram: To gauge overall satisfaction and identify any perceived advantages or disadvantages of the supplemental imaging modalities, participants compared their experience with the imaging procedure to standard mammography. Response options included: “much better”, “better”, “the same”, “worse”, or “much worse”.
- Pain or discomfort during the procedure: To assess physical tolerance and identify procedural aspects affecting patient compliance, participants rated the level of pain or discomfort they experienced on a four-point scale: “none”, “mild”, “moderate”, or “severe”.
- Feelings/emotions during the imaging procedure: Participants described feelings of embarrassment during the procedure to assess discomfort related to physical exposure or body positioning during imaging. Response options were “no”, “minimal”, “moderate” or “severe embarrassment”.
- Fear or anxiety before and during the test: Anxiety levels were rated both before and during the imaging procedure using a five-point scale ranging from “none” to “extreme”. The pre-test question assessed participants’ anticipatory anxiety and its potential effect on their overall experience, while the in-procedure question evaluated their fear or anxiety levels during the test.
- Pre-test information: To evaluate the effectiveness of communication and the informed consent process, participants were asked if they felt adequately informed before consenting to the procedure. Responses were collected in free-text format to encourage detailed feedback.
- Suggestions for improvement: Participants were encouraged to provide recommendations for improving their experience through a free-text question, allowing them to identify specific issues or propose improvements related to the procedures, communication, or logistical arrangements.
- Acceptance: The final question assessed participants’ willingness to undergo the same procedure again, with free-text responses used to capture participants’ reasoning behind their decision.

### Data collection and analysis

Consecutive participants enrolled in the BRAID trial were invited to complete the survey between January and April 2024 during scheduled baseline or follow-up visits without stratification. Questionnaires were administered immediately after imaging to capture participants’ most recent experiences. Completion was entirely voluntary and undertaken independently, without direct assistance or supervision from research staff, to preserve privacy and ensure that responses reflected participants’ own views without bias. Participants were free to omit questions without explanation, and no follow-up was undertaken to obtain data for missing responses.

For each domain, responses from Likert-scale questions were treated as ordinal variables and summarised using descriptive statistics, including response frequencies and percentages or measures of central tendency, where appropriate. Chi-square test was used to assess associations between categorical variables, with statistical significance set at *p* < 0.05. Thematic analysis was employed to analyse free-text responses, identifying recurring themes and patterns in participants’ feedback. Two independent researchers reviewed the responses to ensure the validity and reliability of the findings. Key themes, such as procedural comfort, communication quality, and suggestions for improvement, were integrated into the broader evaluation of patient acceptability.

## Results

During the study period, the questionnaire was offered to *n* = 368 women, of whom 159 returned completed questionnaires, resulting in a response rate of 43.2% (Supplementary Fig. S2). There was no statistically significant difference in response rates between modalities: 48.1% (50/104) for AB-MRI, 45.2% (57/126) for ABUS, and 37.7% (52/138) for CEM (χ^2^ = 2.93, *p* = 0.23). Among 159 respondents, 57/159 (35.8%) were in the ABUS group, 52/159 (32.7%) in the CEM group, and 50/159 (31.4%) in the AB-MRI group (Table 1). Response numbers varied for individual survey questions due to missing responses, with a median completion rate of 81.8 [interquartile range, 79.9–88.3] % among participants.

**Table 1:** Sample size (*n* = 159 women) by imaging modality and timeline

	Supplemental imaging modalities			Total
	AB-MRI	ABUS	CEM	
Baseline (Round 1)	12	6	51	69
18 months (Round 2)	38	51	1	90
Total	50	57	52	159

*AB-MRI* abbreviated breast MRI, *ABUS* automated breast ultrasound, *CEM* contrast-enhanced mammography

### Comparison to standard mammogram

**Fig. 1:**
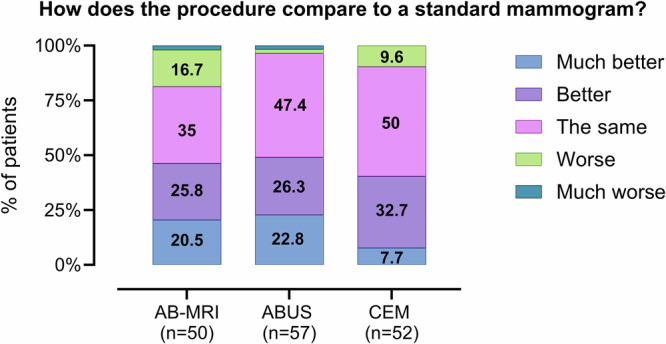
Participants’ evaluations of supplemental imaging modalities in comparison to standard mammography. AB-MRI, abbreviated breast MRI; ABUS, automated breast ultrasound; CEM, contrast-enhanced mammography

All participants (159/159; 100%) responded to this question. Figure 1 summarises participants’ evaluations of supplemental imaging modalities compared to standard mammography. Overall, ABUS and CEM were rated favourably among respondents in these groups, with approximately half (53/109; 48.6%) perceiving them as comparable to standard mammography (ABUS: 27/57, 47.4%; CEM: 26/52, 50%). Additionally, 28/57 (49.1%) ABUS participants and 21/52 (40.4%) CEM participants described these procedures as “better” or “much better” than mammography. In the AB-MRI group, 18/50 (36%) women rated the experience as comparable to mammography, while 13/50 (26%) and 10/50 (20%) considered it as “better” or “much better”, respectively. Among 23 women rating AB-MRI as better than mammography, 14/23 (60.9%) cited reduced pain during imaging due to less breast compression, while 3/23 (13%) preferred it for its higher accuracy in breast cancer detection. The highest proportion of negative feedback (i.e., “worse” or “much worse” than FFDM) was observed for the AB-MRI group (9/50, 18%), followed by CEM (5/52, 9.6%) and ABUS (2/57, 3.5%).

### Pain or discomfort during the procedure

**Fig. 2:**
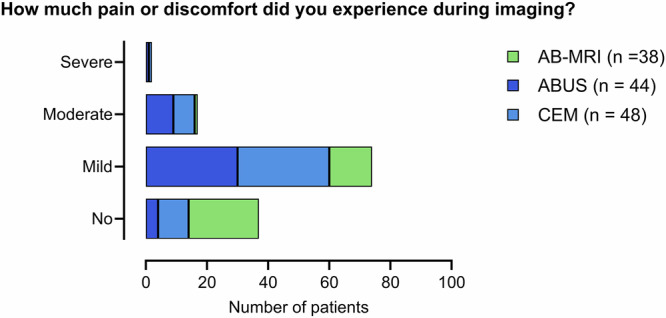
Participant-reported pain or discomfort during the procedure across supplemental imaging modalities. AB-MRI, abbreviated breast MRI; ABUS, automated breast ultrasound; CEM, contrast-enhanced mammography

Information regarding pain or discomfort during imaging was available from 130/159 (81.8%) participants, with 93/130 (71.5%) women experiencing some level of discomfort during imaging. Discomfort levels varied across imaging modalities, with AB-MRI being associated with the least reported discomfort, while higher rates were observed for ABUS and CEM (Fig. 2). In the ABUS group, 40/44 (90.9%) participants experienced mild or severe discomfort, compared to 38/48 (79.2%) CEM participants. while 4/44 (9.1%) reported no discomfort (Fig. 2). Similarly, 38/48 (79.2%) CEM participants experienced mild or severe pain, while 10/48 (20.8%) experienced no discomfort. For AB-MRI, 23/38 (60.5%) participants experienced no discomfort, while 14/38 (36.8%) reported mild or moderate discomfort. Notably, no cases of severe pain or discomfort were reported for AB-MRI (Fig. 2).

### Emotional response during the procedure

Responses were obtained from 126/159 (79.2%) women, most of whom (124/126, 98.4%) reported no or minimal embarrassment during supplemental imaging (Supplementary Table S1). Severe embarrassment was noted by 1/126 (0.8%) participants in the ABUS group, while another participant (1/126, 0.8%) undergoing AB-MRI reported moderate embarrassment.

### Fear or anxiety

**Fig. 3:**
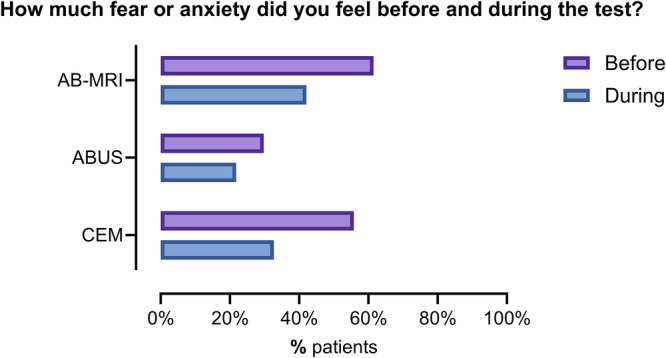
Anxiety levels before and during the procedure across supplemental imaging modalities. AB-MRI, abbreviated breast MRI; ABUS, automated breast ultrasound; CEM, contrast-enhanced mammography

**Fig. 4:**
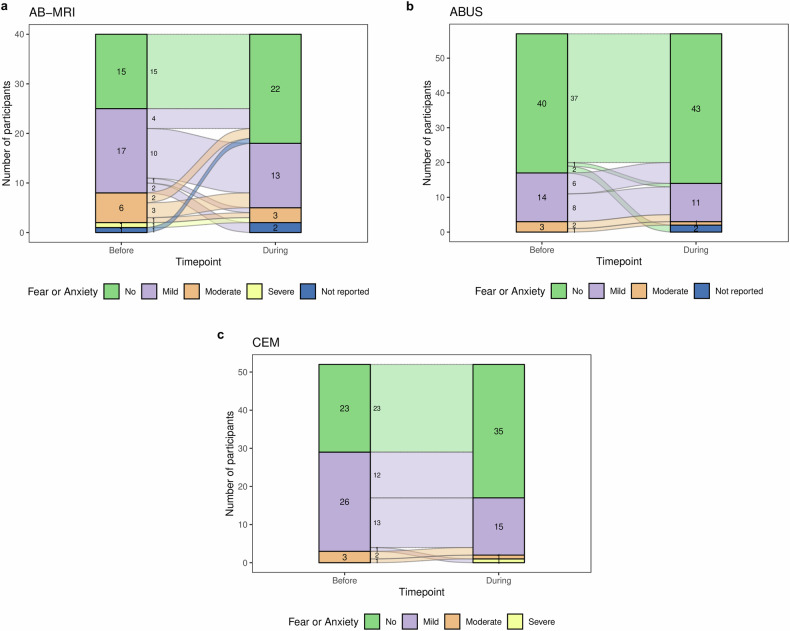
Changes in participants’ anxiety levels before and during the procedure across supplemental imaging modalities. **a** AB-MRI, abbreviated breast MRI; **b** ABUS, automated breast ultrasound; **c** CEM, contrast-enhanced mammography

For this question, responses were available from 144/159 (90.5%) participants. Figure 3 compares fear or anxiety levels before and during the procedure across different supplemental imaging modalities, whereas Fig. 4 illustrates changes in anxiety levels at these timepoints for each modality. Generally, anxiety levels varied by imaging modality, typically remaining the same or decreasing during imaging (Figs. 3, 4). Among 144 women reporting their anxiety levels both before and during imaging, 109/144 (75.7%) noted no change, 32/144 (22.2%) experienced a reduction, and 2.1% (3/144) reported an increase. Participants undergoing AB-MRI had the highest levels of pre-procedural anxiety across the three modalities, with 24/39 (61.5%) experiencing mild-to-severe anxiety before the procedure (Figs. 3, 4a). In comparison, 29/52 (55.8%) and 17/57 (29.8%) women undergoing CEM and ABUS, respectively, experienced mild-to-moderate anxiety before imaging (Fig. 4b, c). Anxiety levels decreased during the procedure for all imaging modalities (Figs. 3, 4). In women undergoing AB-MRI, the proportion reporting no anxiety increased from 38.5% (15/39) before imaging to 57.9% (22/39) during the procedure. In the CEM group, 44.2% (23/52) of participants experienced no pre-procedural anxiety, rising to 67.3% (35/52) during imaging. Similarly, in the ABUS group, the percentage of participants reporting no anxiety increased from 70.2% (40/57) before imaging to 75.4% (43/57) during the procedure (Fig. 4a–c). Participants at follow-up were less likely to report pre-examination anxiety compared to those at baseline (χ^2^ = 5.87, *p* = 0.02), with 50/87 (57.5%) and 15/43 (34.9) reporting fear or anxiety before the procedure for the first and second round of imaging, respectively. For anxiety during the examination, 30/86 (34.9%) baseline participants reported fear/anxiety compared to 11/42 (26.2%) at follow-up (χ^2^ = 0.98, *p* = 0.32).

### Pre-test information and feedback

A total of 130/159 (81.8%) women responded on the adequacy of information received before the examination. Of these, 112/130 (86.2%) felt that the information provided was sufficient, while 18/130 (13.8%) found that aspects of the examination remained unclear. The provision of adequate information was significantly associated with lower pre-procedural anxiety (χ^2^ = 11.4, *p* = 0.01), and more favourable patient ratings of supplemental imaging modalities compared to FFDM (χ^2^ = 29.3, *p* < 0.001). Participants suggested that visual aids, such as infographics, could improve understanding and reduce pre-procedural apprehension.

### Participant feedback

Among the 94/159 (59.1%) participants who provided feedback, two key themes emerged from their free-text responses: their interactions with staff (*n* = 35 responses), and suggestions for enhancing their overall experience (*n* = 18 responses). Positive interactions with staff were noted in 33/35 (94.3%) responses, with 29/35 (82.9%) women reporting that these interactions improved their overall experience. Areas for improvement included logistical challenges, such as short-notice invitations and limited parking facilities. Participant discomfort during MRI scans was primarily due to noise, air quality, and the confined space, which contributed to a less comfortable experience.

### Willingness to repeat the procedure

Responses were received from 130/159 (81.8%) participants, all of whom (*n* = 130, 100%) indicated that they would undergo the supplemental imaging procedure again, reflecting a high level of overall satisfaction and acceptance.

### Withdrawals related to an adverse patient experience

**Fig. 5:**
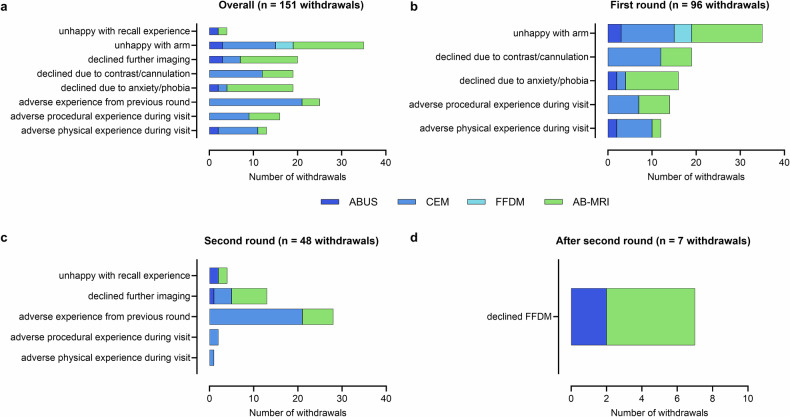
Reasons for patient withdrawals across imaging rounds. **a** Overall withdrawals (*n* = 151); **b** First-round withdrawals (*n* = 96); **c** Second-round withdrawals (*n* = 48); **d** Withdrawals after second round (*n* = 7)

Overall, there were 984 withdrawals in the BRAID trial [6], of which 151/984 (15.3%) were attributed to adverse patient experiences (Supplementary Table S2). Of these 151 withdrawals, the majority involved contrast-enhanced techniques, with 66/151 (43.7%) associated with AB-MRI, and 69/151 (45.7%) with CEM, compared to 12/151 (7.9%) with ABUS (Fig. 5a). Dissatisfaction with the allocated imaging arm accounted for 35/151 (23.2%) withdrawals before the first round of imaging (Fig. 5b; Supplementary Table S3). Within this group, participants most frequently expressed dissatisfaction with being allocated to either AB-MRI (16/35, 45.7%) or CEM (12/35, 34.3%). In the first round, 42 participants withdrew due to adverse physical or procedural experience, including procedure-related anxiety (Fig. 5b; Supplementary Table S3). The vast majority (38/42, 90.5%) of these cases involved contrast-enhanced modalities, with 4/42 (9.5%) related to ABUS. Contrast administration or cannulation concerns were the second most commonly cited reason for experience-related withdrawal in this round, accounting for 19/96 (19.8%) cases (Fig. 5b; Supplementary Table S3). Adverse experiences from the previous round predominated withdrawals in the second round, accounting for 28/48 (58.3%) cases. Among these, 21/48 (43.8%) were associated with CEM, 7/48 (14.6%) with AB-MRI, and none with ABUS (Fig. 5c; Supplementary Table S3). Additionally, of the 13 patients who declined further imaging, 8/13 (61.5%) had previously received AB-MRI, 4/13 (30.8%) CEM, and 1/13 (7.7%) ABUS. Notably, 5/7 (71.4%) patients in the AB-MRI group declined the FFDM examination, which was arranged at the time of their second-round scan (Fig. 5d).

## Discussion

This study highlights the positive reception of three supplemental breast imaging modalities (ABUS, CEM, and AB-MRI) among participants of the BRAID trial, with many considering these techniques comparable or superior to standard mammography, especially in the ABUS and CEM arms. Participants generally perceived these techniques as acceptable and often preferable to mammography, in terms of their diagnostic benefits and overall comfort. These findings are consistent with existing literature [14–16] and support the high level of patient satisfaction associated with supplemental imaging.

When compared to standard mammography, both ABUS and CEM received favourable ratings, with approximately half of participants viewing them as comparable, while a similar proportion considered them superior to FFDM. AB-MRI was also well-received, with several women highlighting benefits, such as reduced pain due to less breast compression and improved detection accuracy. However, AB-MRI was associated with a higher proportion of negative feedback, suggesting that for some patients, its benefits may be outweighed by procedural drawbacks.

In this study, ABUS demonstrated higher acceptance and a more positive patient experience compared to the contrast-enhanced techniques. ABUS has been shown to improve cancer detection in women with dense breasts, while maintaining patient comfort and avoiding contrast-related risks [17, 18]. Its standardised, reproducible imaging reduces operator dependency, while enhancing diagnostic confidence [19], making ABUS a practical option for enhancing breast cancer screening, particularly in resource-limited settings or among patients requiring non-contrast alternatives.

Among contrast-enhanced techniques, our results indicated a general preference for CEM due to faster procedure times, lower anxiety, and greater comfort, although AB-MRI was favoured for breast compression and intravenous contrast sensation. These observations agree with previous studies comparing patient experiences of contrast-enhanced techniques [20, 21] and emphasise that clinical settings and staff interactions play a crucial role in shaping patient perceptions.

Pain and discomfort were significant factors affecting participants’ experiences, with 71.5% of 130 respondents experiencing some level of discomfort. AB-MRI was associated with the least discomfort, with nearly two-thirds of participants reporting no pain. In contrast, mild-to-moderate discomfort was more common with ABUS and CEM, primarily due to breast compression, and in the case of CEM, contrast injection. Severe pain was rare, with only one case reported in each of the ABUS and CEM groups. Emotional responses were largely positive, with almost all participants reporting minimal embarrassment. These findings indicate that while mild discomfort may be a common aspect of supplemental imaging, the procedures are generally well-tolerated by patients.

Fear or anxiety were also important factors, with pre-test anxiety being notably higher among participants undergoing AB-MRI and CEM compared to ABUS. AB-MRI elicited the highest levels of pre-procedural anxiety among imaging methods. However, anxiety levels generally decreased during the procedure. Empathetic and clear communication was key in alleviating anxiety, with many participants emphasising the importance of reassurance and detailed instructions before imaging, particularly for more invasive or complex procedures. Furthermore, women with prior experience of supplemental imaging were less likely to experience anxiety before the examination, while anxiety during the procedure appeared unaffected by prior attendance. When asked if they would undergo the procedure again, all participants expressed willingness, suggesting that supplemental imaging modalities may be well-received in future screening programmes. This is particularly relevant given the evidence supporting the effectiveness of supplemental imaging in enhancing cancer detection in women with dense breasts [22–26].

Withdrawals due to adverse patient experiences were most common for AB-MRI and CEM, highlighting the challenges associated with contrast-enhanced techniques. A significant proportion of withdrawals, many occurring before the first imaging round, were attributed to adverse physical or procedural experiences, dissatisfaction with the allocated imaging arm, or concerns regarding contrast administration or cannulation. ABUS emerged as the most well-tolerated imaging method, with very few withdrawals related to negative experiences. In contrast, withdrawals from AB-MRI were primarily driven by anxiety or contrast-related concerns, while a significant number of CEM withdrawals were due to contrast-agent intolerance. These findings suggest that while contrast-enhanced techniques offer greater accuracy, feasible and safer alternatives like ABUS should be considered, particularly for patients with contraindications or concerns regarding contrast-enhanced modalities.

Overall, a median of 81.8% participants provided responses across questionnaire items. The survey’s focus on personal experiences regarding supplemental imaging may have contributed to incomplete responses across questionnaire items or influenced the pattern of missing responses. Participants likely answered questions they deemed important or relevant to their experience while omitting those they felt were less applicable. Furthermore, factors such as limited personal time, distractions or noise, particularly since questionnaires were typically completed in a clinical environment after the procedure, or fatigue following imaging may have affected completion rates. Notably, the lowest response rate was observed for free-text questions (59.2%), potentially reflecting the additional time or effort required to provide written feedback.

While this study provides valuable insights, its limitations include a single-centre design, a relatively small sample size, and the opportunistic nature of this investigation, which was not a pre-specified outcome of BRAID. These factors may have restricted our ability to capture the full spectrum of patient experiences. The absence of an a priori sample size calculation represents an additional limitation, as the cohort size was emergent and determined by participant attendance during the survey period and willingness to complete the questionnaire rather than a predefined recruitment target. While this approach was appropriate given the descriptive objectives and opportunistic nature of our study, as well as the lack of prior data on effect size to guide sample size estimation, it may have limited the precision and generalisability of our results.

Data collection occurred during scheduled imaging appointments without stratification (i.e., baseline or follow-up), with questionnaire completion being entirely voluntary. Thus, the distribution of participant responses across imaging modalities and rounds likely reflected various factors, including the timing of imaging rounds, participant attendance patterns and willingness to complete the questionnaire, rather than stratified sampling. These factors, together with the fact that participants voluntarily participated in both the NHSBSP and the BRAID trial, may have led to potential selection bias. The voluntary nature of questionnaire completion, with 43.2% of those invited responding, may also have introduced self-selection bias, as respondents might have differed systematically from non-respondents. This relatively low overall response rate, combined with limiting data collection to participants attending imaging appointments during a predefined period, may have reduced the representativeness of our sample and limited the generalisability of our findings to the broader screening population. Nonetheless, among respondents, a median of 81.8% or more provided answers across questionnaire items, which supports the reliability of our results and helps reduce the potential impact of non-response bias. Our study highlights important areas of patient experience that could inform future screening strategies. Future research should involve larger, multicentre trials to evaluate how factors such as age, ethnicity, and prior imaging history influence patient experiences with breast imaging.

In summary, our results suggest that supplemental imaging modalities (ABUS, CEM, and AB-MRI) are generally well-received. Important factors in promoting acceptance are clear communication, empathetic care, and patient education. Addressing psychological barriers and prioritising patient comfort will be essential for integrating supplemental imaging into routine breast cancer screening. These findings support a shift from a “one-size-fits-all” approach to a more tailored screening strategy, improving outcomes for women with dense breasts.

## Supplementary information

Supplementary information

## Data Availability

Access to the data supporting this study may be granted upon reasonable request to the Principal Investigator (fjg28@cam.ac.uk) and may require a data sharing agreement.

## Abbreviations

AB-MRI: Abbreviated breast magnetic resonance imaging
ABUS: Automated whole-breast ultrasound
BI-RADS: Breast Imaging Reporting and Data System
BRAID: Breast screening: risk adaptive imaging for density
CDR: Cancer detection rate
CEM: Contrast-enhanced mammography
FFDM: Full-field digital mammography
MRI: Magnetic resonance imaging
NHSBSP: National Health Service Breast Screening Programme
UK: United Kingdom
