# Supplementary material for: Acceptance, experience, and feedback for supplemental screening in dense breasts among women participating in the BRAID trial
Source: Insights Imaging. 2026 Jan 16;17:14. doi: 10.1186/s13244-025-02170-8 (PMC12811222; doi:10.1186/s13244-025-02170-8)
Supplement: Supplementary file 1 — Supplementary information [file 13244_2025_2170_MOESM1_ESM.pdf]

Acceptance, experience and feedback for supplemental screening in dense Breasts among women participating in the BRAID trial

ELECTRONIC SUPPLEMENTARY MATERIAL

Supplemental Figure S1: BRAID (Breast screening: Risk Adaptive Imaging for Density trial) flowchart. AB-MRI: abbreviated breast MRI; ABUS: automated breast ultrasound; CEM: contrast-enhanced mammography; FFDM: Full-field digital mammography; BIRADS: Breast Imaging Reporting and Data System

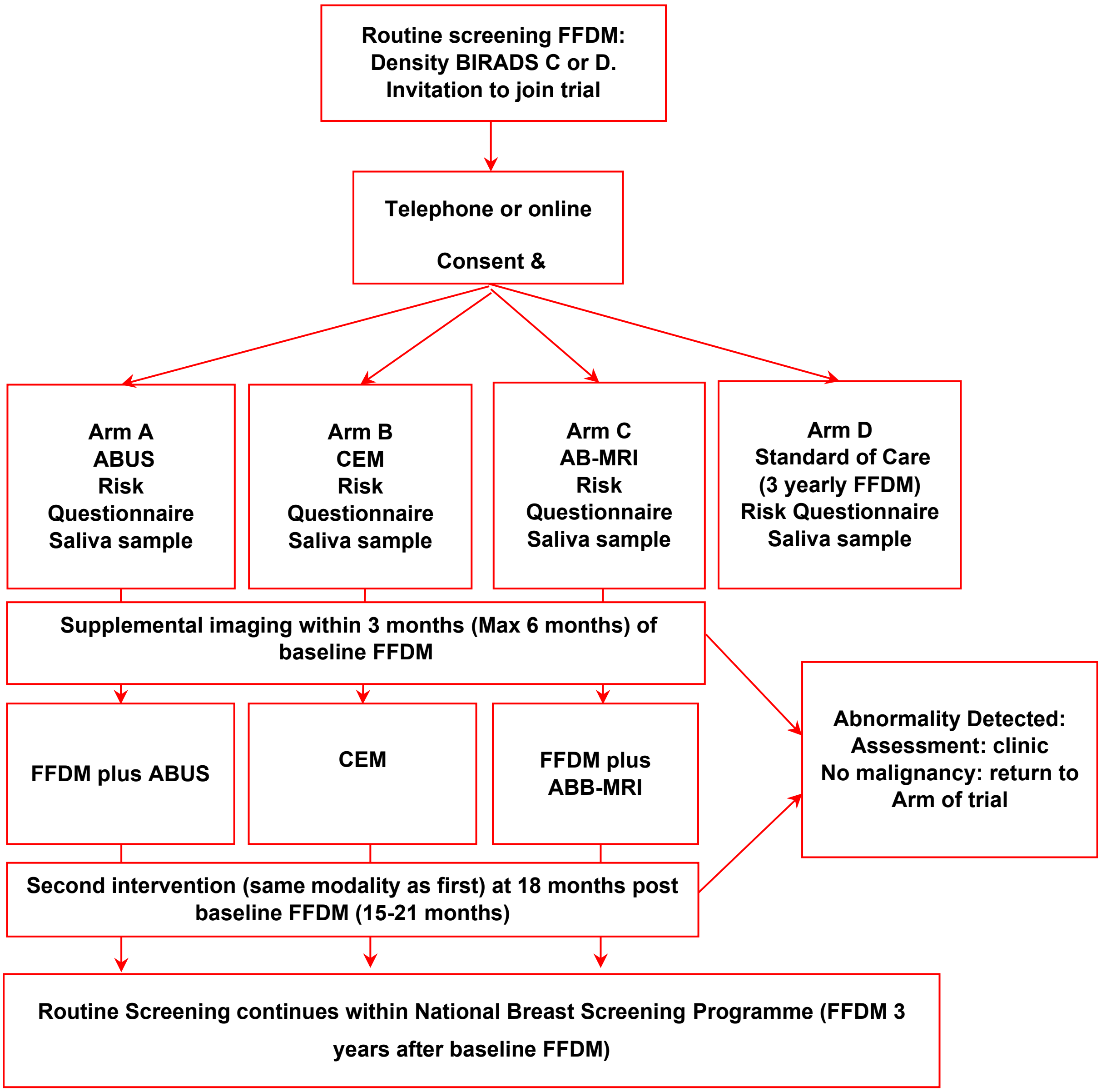

Supplemental Figure S2: Questionnaire distribution and response numbers by supplemental imaging modality and screening round.

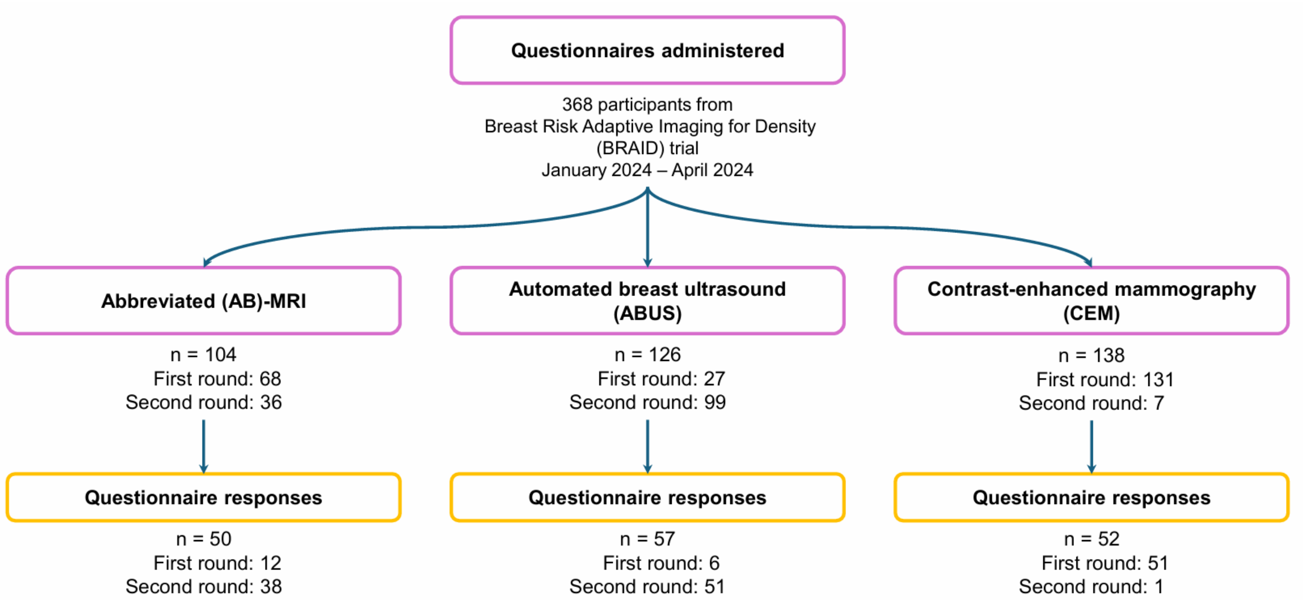

Supplemental Table S1: Self-reported levels of embarrassment across supplemental imaging modalities (n = 126 women)

Embarrassment level	Supplemental imaging modalities			*Total*
	**AB-MRI**	**ABUS**	**CEM**	
No	34	36	44	110
Mild	3	7	4	14
Moderate	1	0	0	1
Severe	0	1	0	1
Total	38	44	48	**126**

AB-MRI: abbreviated breast MRI; ABUS: automated breast ultrasound; CEM: contrast-enhanced mammography

Supplemental Table S2: Reasons for patient withdrawals (n = 984) in the Breast Screening: Risk Adapted Imaging for Density (BRAID) trial.

**Withdrawals – Overall (n=984 patients)**	**n (%)**
Patient experience	151 (15.3)
Contraindications to imaging[a]	38 (3.8)
Ineligible	32 (3.3)
Lost to follow-up	201 (20.4)
Unable to attend imaging	196 (19.9)
No reason given	176 (17.9)
Other[b]	190 (19.9)

[a]Contraindications to imaging modality or contrast.

[b]Reasons including lack of imaging capacity at site, trial procedural issues/protocol adherence, participant opting for other research studies.

Supplemental Table S3: Patient withdrawals related to patient experience stratified by imaging round.

Withdrawals – First round (n = 96 patients)	n (%)
Adverse physical experience during visit[a]	12 (12.5)
Adverse procedural experience during visit[b]	14 (14.6)
Declined due to anxiety/phobia[c]	16 (16.7)
Declined due to contrast/cannulation[d]	19 (19.8)
Unhappy with imaging arm	35 (36.5)
**Withdrawals – Second round (n = 48 patients)**	**n (%)**
Adverse physical experience during visit	1 (2.1)
Adverse procedural experience during visit	2 (4.2)
Adverse experience from previous round[e]	28 (60.9)
Declined further imaging	13 (27.1)
Unhappy with recall experience	4 (8.3)
**Withdrawals – After second round (n = 7 patients)**	**n (%)**
Declined FFDM	7 (7)

[a]Adverse physical experience during imaging due to patients feeling faint or fainting during the visit, experiencing minor allergic reactions, visit or feeling pain during imaging
[b]Adverse procedural experience during visit due to technical issues during the imaging session causing discomfort, including repeated imaging, extravasation of contrast, or multiple cannulation attempts.
[c]Participants declining due to anxiety about the examination, exam results, fear of the modality, including claustrophobia if MRI.
[d]Withdrawal reasons included needle phobia or contrast concerns.
[d]Only relevant for participants who completed round 1, but experienced issues, including contrast reactions or claustrophobia.
FFDM: full-field digital mammography.

BRAID Study Participant Experience Survey

Thank you so much for your time and support for the BRAID Study.
We would like to hear your feedback and seek ways to improve our service.
Please use the back of the paper if you run out of space.

Trial ID:________________ Procedure:________________

1. Compared to the screening mammogram, was the procedure you received today
    - ✈ Much better
    - ✈ Better
    - ✈ The Same
    - ✈ Worse
    - ✈ Much worse In what ways?

2. If this procedure is offered to you again, would you have it? What could be done to improve your experience?

3. Before consenting, did you feel you received enough information and know what to expect during the procedure?

4. Based on what you knew about this particular test, how much fear or anxiety did you have about having the test **before** the test?
    - No fear or anxiety
    - Mild fear or anxiety
    - Moderate fear or anxiety
    - Severe fear or anxiety
    - Extreme fear or anxiety

5. Thinking about this test, how much pain or discomfort did you experience **while it was happening**?
    - No pain or discomfort
    - Mild pain or discomfort
    - Moderate pain or discomfort
    - Severe pain or discomfort
    - Extreme pain or discomfort
6. How much embarrassment, if any, did you actually feel during the test experience (for example, revealing parts of your body, movements of the body or other aspects that may have caused you to feel awkward or uncomfortable)?
    - No embarrassment
    - Mild embarrassment
    - Moderate embarrassment
    - Severe embarrassment
    - Extreme embarrassment
7. How much fear or anxiety did you feel during this test experience?
    - No fear or anxiety
    - Mild fear or anxiety
    - Moderate fear or anxiety
    - Severe fear or anxiety
    - Extreme fear or anxiety
8. Is there anything else you would like to share with us?

BRAID Study Participant Experience Survey

Thank you so much for your time and support for the BRAID Study.
We would like to hear your feedback and seek ways to improve our service.

Please select **‘NHS Wi-Fi’** on your mobile device and scan the QR code below.

Or, access through the link:
https://forms.office.com/e/TwpKCkFFFH

We also have paper copies available; just let us know~

Ask the trial team for your randomisation ID and imaging arms. All data collected are anonymised.

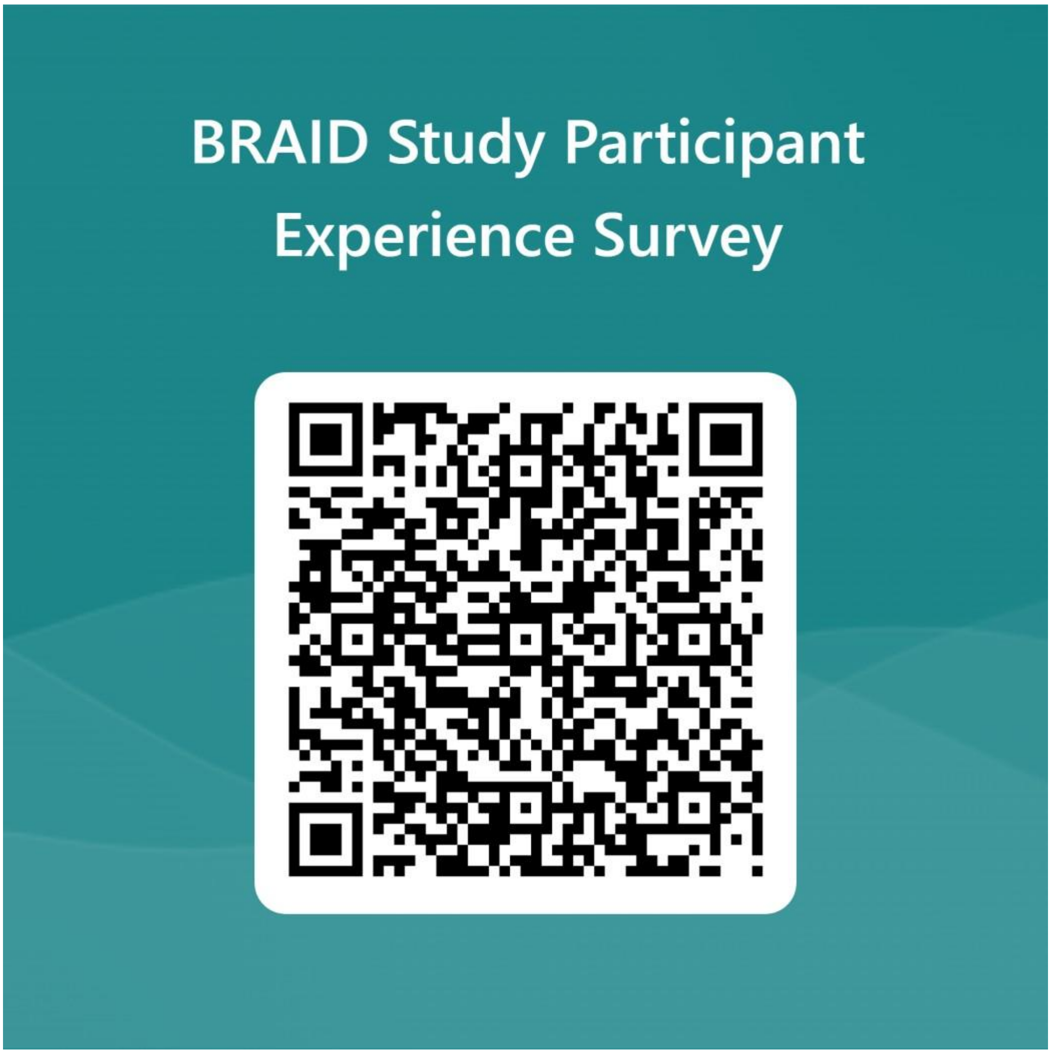
BRAID Study Participant
Experience Survey
